# The effects of chemical fungicides and salicylic acid on the apple microbiome and fungal disease incidence under changing environmental conditions

**DOI:** 10.3389/fmicb.2024.1342407

**Published:** 2024-02-05

**Authors:** Michael S. McLaughlin, Svetlana N. Yurgel, Pervaiz A. Abbasi, Shawkat Ali

**Affiliations:** ^1^Kentville Research and Development Centre, Agriculture and Agri-Food Canada, Kentville, NS, Canada; ^2^Department of Plant, Food, and Environmental Sciences, Faculty of Agriculture, Dalhousie University, Truro, NS, Canada; ^3^United States Department of Agriculture (USDA), Agricultural Research Service, Grain Legume Genetics and Physiology Research Unit, Prosser, WA, United States

**Keywords:** chemical fungicides, salicylic acid, *Malus domestica*, microbiome, environmental conditions

## Abstract

Epiphytic and endophytic micro-organisms associated with plants form complex communities on or in their host plant. These communities influence physiological traits, development, and host susceptibility to abiotic and biotic stresses, and these communities are theorized to have evolved alongside their hosts, forming a unit of selection known as the holobiont. The microbiome is highly variable and can be influenced by abiotic factors, including applied exogenous agents. In this study, we compared the impact of chemical fungicide and salicylic acid treatments on the fungal communities of “Honeycrisp” apples at harvest over two consecutive growing years. We demonstrated variations in fungal community structure and composition by tissue type, growing season, and treatment regimes and that fungicide treatments were associated with reduced network complexity. Finally, we show that the inclusion of salicylic acid with 50% less chemical fungicides in an integrated spray program allowed a reduction in fungicide use while maintaining effective control of disease at harvest and following storage.

## Introduction

Domesticated apples (*Malus* × *domestica* Borkh) are a globally consumed commodity with rapidly growing demand and production. From 2010 to 2020, apple production increased by over 20%, from 71.19 million metric tonnes to 86.44 million metric tonnes, and the apple market is expected to grow at an average compound annual growth rate of 4% each year from 2019 to 2027 (Shahbandeh, 2022). Pre- and post-harvest diseases, primarily caused by fungi, can lead to significant losses of yield and quality, with post-harvest fungal pathogens estimated to cause losses of 30–40% in developing countries (Patriarca, 2019). For decades, these fungal pathogens have primarily been managed with chemical fungicides, especially since modern orchards have an expected lifespan of 2–3 decades, which eliminates the possibility of quickly bringing in new resistant varieties (Kelman, 1989). However, in recent years, the use of chemical fungicides has become increasingly restricted due to concerns about human and environmental health, leading to the de-registration of several of these products (Ragsdale and Sisler, 1994; Steinhauer et al., 2018). Consequently, growers and researchers are constantly searching for new, more eco-friendly options in order to protect vulnerable apple fruit in a shifting regulatory landscape.

Plants have evolved in close association with their respective microbial communities (Krings et al., 2007; Delaux and Schornack, 2021), and evidence suggests that this process continued in apple throughout the domestication of this species (Abdelfattah et al., 2021b). Plant-associated microbial communities can have an immense impact on the features of their host, including susceptibility/resistance to pre- and post-harvest diseases, drought stress tolerance, yield, and fruit quality (Berg et al., 2015). Therefore, alterations to the microbial community can have significant ramifications for the health of the host plant. Indeed, the development of disease is sometimes associated with microbiome dysbiosis and can be correlated with a loss in microbial diversity. For example, in mango, microbiome diversity has been correlated with susceptibility to stem-end rot caused by *Alternaria alternata*, and mango fruit characterized as having lower diversity (such as green fruit or fruit stored at lower temperatures) were consistently more susceptible to disease (Diskin et al., 2017). Similarly, the incidence of *Fusarium* wilt disease of tomato is negatively correlated with the bacterial and fungal diversity of rhizosphere communities (Zhou et al., 2022). More recently, it has been suggested that actively maintaining the microbiome in a “healthy state” could be a viable, environmentally friendly method of disease control, particularly for post-harvest pathogens (Berg et al., 2020). Alternatively, tailored microbial communities could be designed to improve disease resistance (Ali et al., 2023). However, in order to achieve these goals, a thorough understanding of individual crop microbiomes is necessary, including how these microbiomes responds to various biotic and abiotic factors, as well as how these changes can impact disease development.

While the rhizosphere and phyllosphere of apple have been extensively studied, the factors impacting the microbiome of apple fruit are not well understood (Whitehead et al., 2021; Abdelfattah et al., 2021a). Recently, we demonstrated that variations in environmental conditions between growing seasons had the greatest influence on the apple fruit microbiome compared to tissue type, management practice, and geographical location within a single ecozone (McLaughlin et al., 2023). However, compelling evidence suggests that many factors—including geographical location, management practices, tissue type, cultivar., storage duration, and pre-storage treatments—can significantly impact the structure and diversity of the fungal microbiome of apple fruit (Abdelfattah et al., 2016; Bösch et al., 2021; Britt et al., 2021; Abdelfattah et al., 2021a). Though interaction effects between the geographical location and tissue type have been reported (Abdelfattah et al., 2021a), determining how all of the aforementioned factors interact with one another to influence the apple fruit microbiome still requires more investigation.

In contrast to conventional chemical fungicides, plant defence elicitors such as salicylic acid (SA) increase natural plant immunity through the stimulation of systemic acquired resistance, thereby priming the plant for subsequent pathogen infections (Bektas and Eulgem, 2015). This naturally occurring phytohormone is rapidly metabolized by apples, resulting in no traces of elevated SA in apple fruits within 7 days of exogenous application (da Rocha Neto et al., 2016). This is in stark contrast to chemical fungicides, which may leave chemical residues in the fruit for longer periods (Ragsdale and Sisler, 1994). Therefore, plant defence elicitors such as SA are believed to be a more “eco-friendly” form of disease management than chemical fungicides (Wang et al., 2015). Since plant defence elicitors are not toxic to microorganisms, they might also be less disruptive to the microbiome compared to chemical fungicides. At the same time, it has been previously shown that the endogenous expression of the SA phytohormone has repercussions for the host microbiome. Differential representation of several bacterial families was found in the root microbiome of *Arabidopsis* SA knock-out mutants (Lebeis et al., 2015). In addition, the bacterial phyllosphere communities of *Arabidopsis* mutants exhibiting elevated SA signalling were characterized by increased diversity (Vincent et al., 2022). Given that changes in the level of endogenous SA can significantly alter the microbiome, it is possible that exogenous applications of this phytohormone may have similar ramifications.

Plant defence elicitors, though initially seen as a promising alternative to chemical fungicides, are generally less effective than fungicides in the field (Cao et al., 2006). However, including plant defence elicitors in conventional fungicide spray programs could still potentially reduce the use of conventional fungicides and thus the negative impacts of these treatments on the plant microbiome. While a number of studies have compared the impacts of conventional and organic management practices on the fungal and bacterial communities of apple fruit, these studies focus primarily on chemical fungicides (Abdelfattah et al., 2016; Wassermann et al., 2019), and thus the impact of plant defence elicitors on the microbiome is a weak point in the literature.

In this study, we characterize the fungal microbiome of untreated “Honeycrisp” apples and compare the impact of conventional fungicides, SA, and an integrated program (50% less conventional fungicides plus SA) on apple fungal communities and disease prevalence. In addition, we explore the impact of conventional fungicides on fungal community networks. Lastly, we examine how different environmental conditions (growing season) might interact with various tissue types and treatments in impacting fungal microbiota. To achieve this goal, the different treatment regimes were compared using fungal alpha-diversity metrics, the presence of differentially represented genera and unique amplicon sequence variants (ASVs), and co-occurrence networks.

## Materials and methods

### Site description and treatments

The field experiment was carried out in a mature orchard using model apple cultivar “Honeycrisp” at the Kentville Research and Development Centre (KRDC) research farm (45° 04′ 08″ N 64° 28′ 41″ W) during two growing seasons (years 2019 and 2020). Each year, 64 apple trees were divided into four blocks of 16 trees. Four treatments, i.e., (i) conventional (chemical fungicides), (ii) integrated (chemical fungicides and SA), (iii) SA alone, and (iv) control (untreated), were compared in a randomized complete block design with four replicates (Supplementary Figure S1). The SA treatment consisted of 12 (2019) and six (2020) foliar sprays of 1-mM SA during the growing season, while the conventional treatment consisted of 12 foliar sprays of chemical fungicides during both growing seasons. The integrated treatment consisted of a combination of six applications of a 1-mM SA spray and six separate applications of chemical fungicides each year.

### Plant material

For the purposes of microbial analysis, five individual fruits per tree were sampled at commercial maturity from two healthy trees per treatment per in late September of 2019 and early October of 2020, respectively. The sampled trees consisted of the middle two trees in each treatment’s biological replicate, in order to reduce drift from neighbouring treatment groups. To preserve epiphytes, no surface sterilization was carried out. To collect the peel samples, 3 cm of peel from the circumference of the equator was removed using a sterile peeler. An apple corer was used to remove the apple core tissues, and 3 cm of the stem and calyx ends were used for DNA extraction. All the apple tissues were then flash-frozen in liquid nitrogen. The peel and core tissues from five individual fruits from the same tree were combined to make a single biological replicate (for a total of eight biological replicates per treatment, per tissue), which was freeze-dried using a lyophilizer at −80°C for 24 h and 3 days, for the peel and core tissues, respectively, before being ground into a powder using a mortar and pestle. Subsequently a QIAGEN PowerSoil^®^ DNA Isolation Kit (Cat. No. 12888-100) was used to isolate the fungal DNA, following the manufacturer’s protocol.

To determine disease incidence at harvest, 40–50 apple fruit per tree were selected at commercial maturity from two trees per treatment per block using the same method used in the microbiome sampling, and were visually examined for the incidence of apple scab, black rot, and bitter rot. An additional twenty asymptomatic apples per tree were selected to undergo cold storage at 3°C for a period of 4 months before being visually examined for post-harvest disease. Significance of treatment differences were calculated based on a student *t*-test (*p* < 0.05).

### PCR amplification of target DNA and Illumina sequencing

The fungal internal transcribed spacer (ITS) region was amplified using the fungal-specific ITS primers ITS3/KYO2 (Toju et al., 2012) and ITS4 (White, 1990), with the addition of a blocking oligo (50-ATTGATATGCTTAATTCAGCGGGTAACCCCGCCTGACCTGGGGTC-GCGTT-C3 spacer 30) (Abdelfattah et al., 2021a) in order to prevent the amplification of host DNA. The PCR reaction was identical to that in our previous study (McLaughlin et al., 2023). A ~ 350-bp band was obtained by running the PCR product on a 0.5% agarose gel, and subsequently excised and purified using a QIAGEN gel extraction kit (QIAEX II^®^ Gel Extraction Kit, Cat. No. 20051) following the manufacturer’s protocol. Next, 10 μL of the purified DNA was sent to the Dalhousie University CGEB-IMR Laboratory^1^ for Illumina sequencing using the reference primers ITS86F (5’-GTGAATCATCGAATCTTTGAA-3′) and ITS4(R) (5′- TCCTCCGCTTATTGATATGC-3). Sequences were multiplexed using an Illumina MiSeq platform and dual indexing to generate 2 × 300 bp paired-end reads. The Illumina sequencing and multiplexing was conducted as described in Comeau et al. (2017). The raw sequencing data is publicly available here https://www.ncbi.nlm.nih.gov/sra/PRJNA1021219.

### Bioinformatics and statistical analysis

Stitched paired-end reads were first generated from raw amplicon sequences using the PEAR paired-end read merger (Zhang et al., 2014). The primer sequences were then trimmed from the available reads using Cutadapt v1.11 (Martin, 2011). Further processing of trimmed reads was conducted using QIIME 2, version 2022.8 (Bolyen et al., 2019), and reads were trimmed to a standard length of 261 bp using the QIIME 2 plugin q2-deblur. The taxonomic classification of amplicon sequence variants (ASVs) was carried out by using the naïve Bayesian classifier RDP Classifier r and the UNITE ITS database v7.2 (Nilsson et al., 2019). ASVs with a mean relative abundance of less than 0.01% of the dataset were removed in order to account for the Illumina platform’s sequencing error rate, after which plant-derived ASVs (ex: chloroplasts) were also removed.

The relative abundance of individual features, alpha and beta-diversity indices, and core taxa (ASVs present in 75% of all samples) were calculated using QIIME 22022.8. ADONIS tests were also conducted in QIIME 2 using a weighted Bray Curtis dissimilarity in order to determine the degree of variance that could be explained by different variables. Non-metric multi-dimensional scaling (NMDS) analyses of fungal communities, as well as Venn diagrams based on individual ASVs, were constructed in R (v4.2.3, https://R-project.org/). Similarly, ALDEx2 v1.24.0 was used in R to determine differentially represented features between groups, and the significance of the results (*p* < 0.05) were assessed by performing a Benjimini-Hochberg multiple test correction of a Welch’s *t*-test (tissue, year) or Kruskal-Wallis test (treatments) (Fernandes et al., 2013). The CCREPE (Compositionality Correct by Renormalization and Permutation) R package (Schwager et al., 2014) was used to perform co-occurrence analysis. The four treatments were divided into two separate networks based on fungicide use: a non-fungicide network, which consisted of the untreated control and SA-treated samples, and a fungicide network, which consisted of the samples treated with chemical fungicide and integrated treatments. A total of 40 samples (20 per treatment (5 per year per tissue)) were used to explore the microbial networks at the species level. For each network, interactions with *p* values >0.05 were removed from the dataset, and the network data was visualized using Cytoscape (Shannon et al., 2003). In the network visualization, the nodes represent individual taxa while the edges represent positive (red) and negative (blue) interactions between nodes. Networks were visualized as an “edge-weighted spring-embedded” co-occurrence network.

## Results

### General structure of apple fungal microbiota

After removing host sequences and amplicon sequence variants (ASVs) with a mean relative abundance of less than 0.01% of the total relative abundance (TR), 3,500,302 reads were retained in 128 samples analyzed in this study. A total of 209 unique ASVs corresponding to two fungal phyla, 18 classes, 39 orders, 74 families and 115 genera were identified. *Ascomycota* (94% of TR) was the most prevalent fungal phyla in terms of relative abundance, followed by *Basidiomycota* (6% of TR). The most abundant classes included *Dothideomycetes* (71% of TR), followed by *Eurotiomycetes* (21% of TR) and *Microbotryomycetes* (5% of TR). The most relatively abundant classes included *Dothideomycetes* (71% of TR), followed by Eurotiomycetes (21% of TR) and *Microbotryomycetes* (5% of TR). The *Dothideomycetes* were primarily represented by the orders *Capnodiales* (35% of TR), *Dothideales* (26% of TR)*, and Pleosporales* (10% of TR), while *Eurotiomycetes* and *Microbotryomycetes* were represented by the orders *Eurotiales* (21% of TR) and *Sporidiobolales* (5% of TR), respectively. Finally, the most abundant genera in the dataset included *Cladosporium* (35% of TR), *Aureobasidium* (26% of TR), *Penicillium* (21% of TR), *Alternaria* (7% of TR) and *Sporobolomyces* (5% of TR); together, these five genera accounted for approximately 94% of total relative abundance. A full breakdown of resolved taxa from the dataset are presented in Supplementary Table S1.

### The fungal microbiome of untreated core and peel tissues

**Figure 1:**
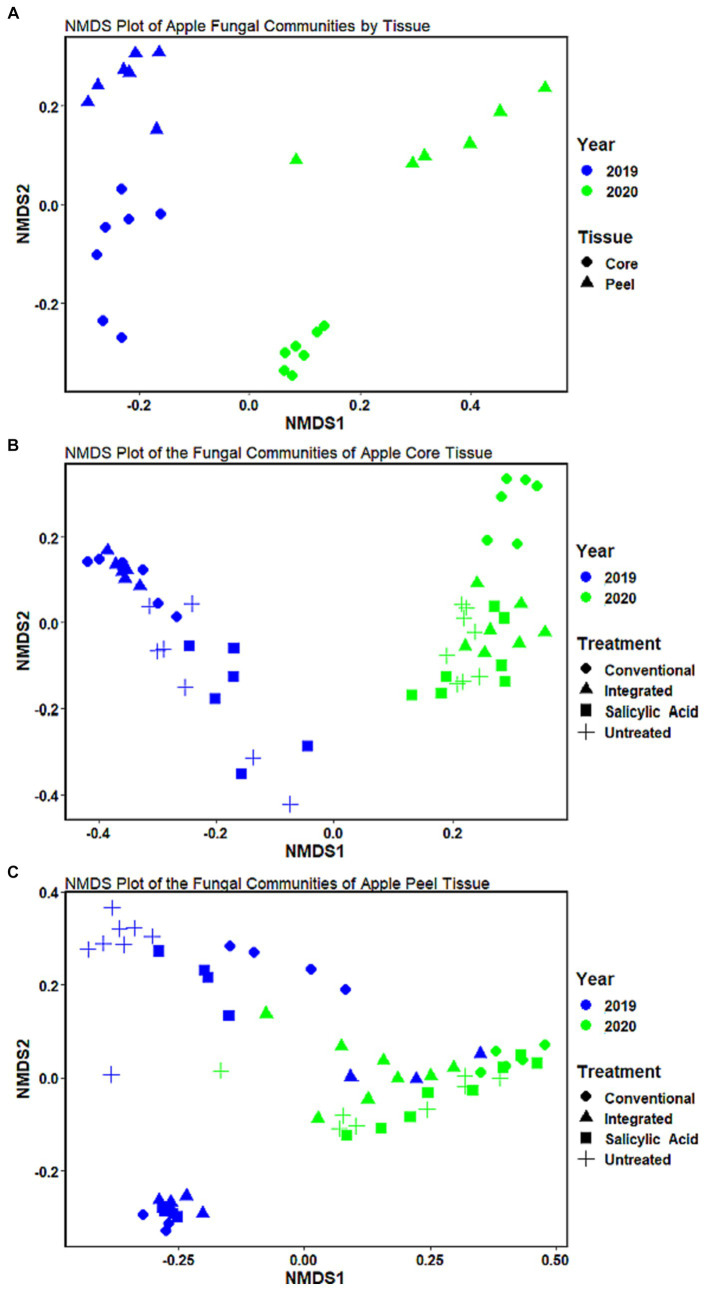
Non-metric multidimensional scaling (NMDS) of the fungal communities of Honeycrisp apples at the ASV level. **(A)** The communities in untreated tissues clustered by growing season and tissue type. **(B)** The communities in core tissues clustered by treatment regime and growing season. **(C)** The communities in peel tissues **(C)** clustered by treatment regime and growing season.

**Figure 2:**
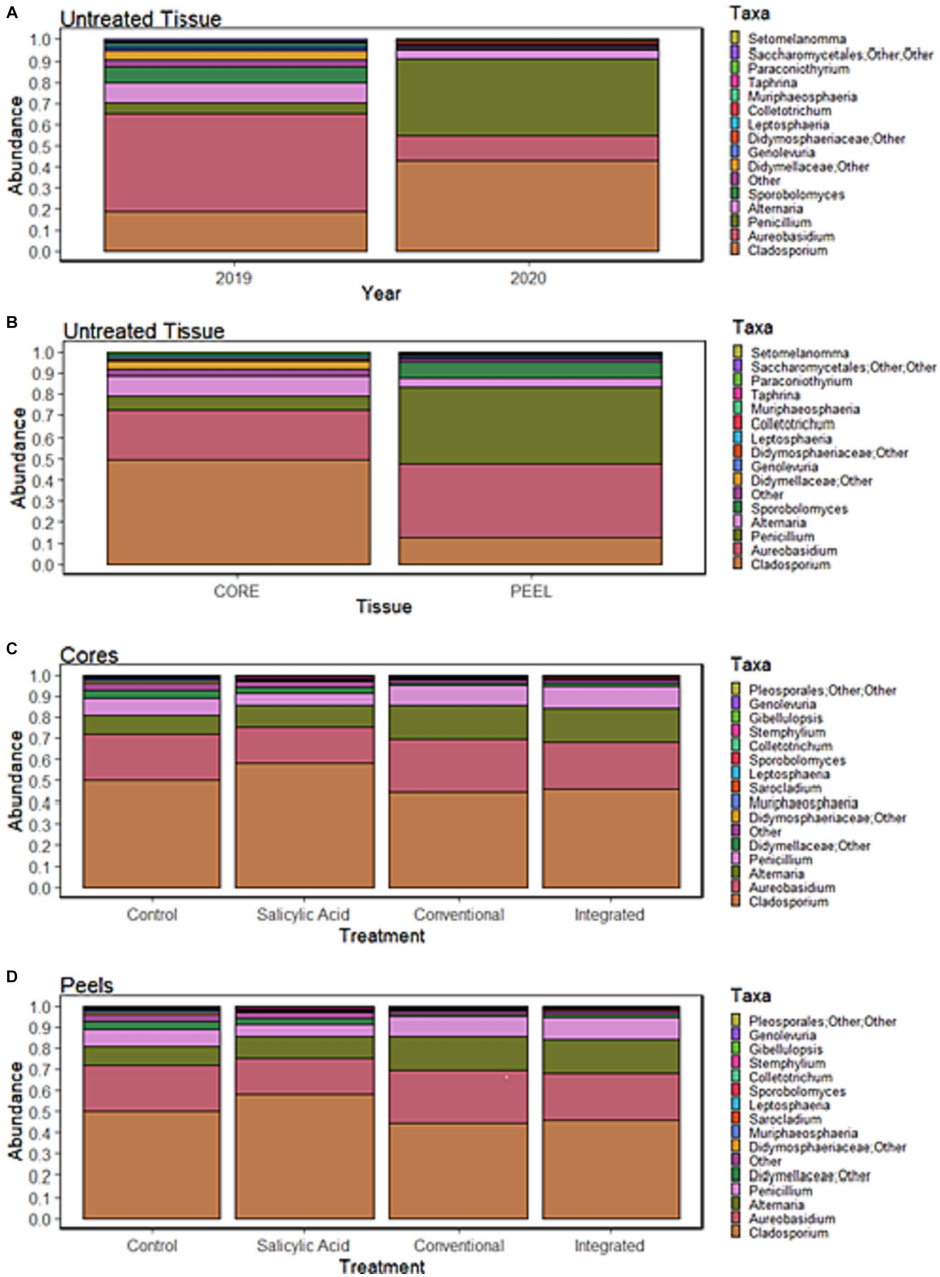
Relative abundance of the top 15 most abundant genera in Honeycrisp apples. **(A)** Communities in samples of untreated tissue grouped by growing season. **(B)** Communities in samples of untreated tissue grouped by tissue type. **(C)** Communities in samples of core tissues grouped by treatment regime. **(D)** Communities in samples of peel tissues grouped by treatment regime.

**Figure 3:**
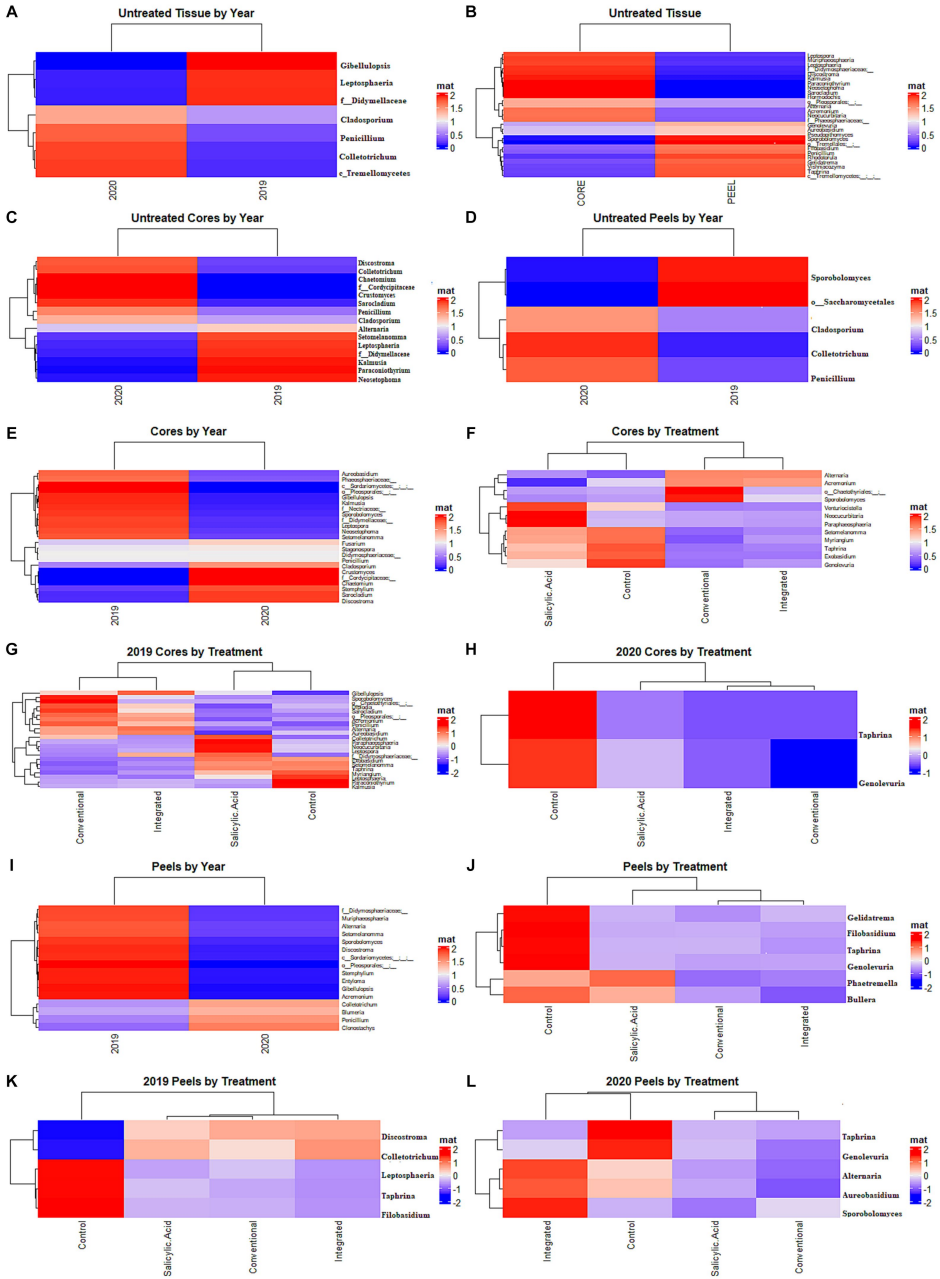
Differential representation of fungal genera. **(A)** Samples of untreated tissues grouped based on growing season. **(B)** Samples of untreated tissues grouped based on growing season. **(C)** Samples of untreated core tissues grouped based on growing season. **(D)** Samples of untreated peels grouped based on growing season. **(E)** Samples from treated core tissues grouped based by growing season. **(F)** Samples from core tissues grouped by treatment regime. **(G)** Samples from core tissues grouped by treatment in 2019. **(H)** Samples from core tissues grouped by treatment in 2020. **(I)**, Samples from treated peels grouped based by growing season. **(J)** Samples from peels grouped by treatment regime. **(K)** Samples from peels grouped by treatment in 2019. **(L)** Samples from peels grouped by treatment in 2020. Statistical significance was calculated using a Benjamini-Hochberg multiple test correction of a Welch’s *t*-test (growing season) or Kruskal-Wallis test (treatment) in Aldex 2 (*p* < 0.05).

To investigate the effect of tissue type on fruit microflora, we focused on the fungal microbiome of untreated control samples. The environmental conditions during the growing season appeared to have the greatest impact on community structure (R2 = 0.42, *p* < 0.001), followed by tissue type (R2 = 0.31, *p* < 0.001). A small but significant interaction effect was observed between tissue type and growing season (R2 = 0.07, *p* < 0.001), indicating that the growing season might have a different impact on fungal communities depending on the tissue type. NMDS visualizations revealed a clear separation of communities with samples clustering by growing season (Figure 1A). Untreated 2019 tissue samples had a much higher Shannon diversity index (*p* < 0.05) than of 2020 (Table 1). In untreated tissue, communities were dominated by a small number of genera, with the15 most abundant genera accounting for over 96 and 98% of the TR in 2019 and 2020, respectively (Figure 2A). In the communities associated with untreated tissue, seven genera were differentially represented by growing season, most being more abundant in 2020 (Figure 3A). These genera included several abundant taxa, such as *Cladosporium* (19 and 43% of TR in 2019 and 2020, respectively) and *Penicillium* (56 and 36% of TR, respectively).

**Table 1:** Apple fungal community Shannon diversity.

**Year (untreated)**	
	Shannon Diversity (SD)
2019	2.56A (0.41)
2020	2.04B (0.58)
**Tissue type (untreated)**	
	Shannon Diversity (SD)
Core	2.66A (0.31)
Peel	1.94B (0.51)

Core tissue	
**Growing season (untreated core tissue)**	Shannon Diversity (SD)
2019	2.66A (0.31)
2020	1.94B (0.51)
**Growing season (treated core tissue)**	Shannon Diversity (SD)
2019	2.79A (0.29)
2020	2.16B (0.38)
**Core tissue by treatment (2020)**	Shannon Diversity (SD)
Untreated	2.52A (0.18)
Salicylic acid	2.27AB (0.23)
Conventional	1.66C (0.33)
Integrated	2.12 BC (0.19)

Peel tissue	
**Growing season (untreated peel tissue)**	Shannon Diversity (SD)
2019	2.27A (0.27)
2020	1.66B (0.45)
**Growing season (treated peel tissue)**	Shannon Diversity (SD)
2019	2.54A (0.57)
2020	1.47B (0.52)
**Peel tissue by treatment (2020)**	Shannon Diversity (SD)
Untreated	1.65AB (0.46)
Salicylic acid	1.36AB (0.51)
Conventional	0.89B (0.49)
Integrated	1.76A (0.3)

Shannon diversity index values for apple tissues (*p* < 0.05). Standard deviation (SD) is given in brackets.

In the untreated samples, the fungal communities in both core and peel tissues formed distinct clusters when visualized with NMDS (Figure 1A). In addition, the communities associated with the untreated core tissue had significantly higher Shannon diversity (*p* < 0.05) than those associated with untreated peel tissue (Table 1). The 15 most abundant genera in the untreated tissue had a total relative abundance of over 95 and 98% of the TR in core and peel tissue, respectively (Figure 2B). A total of 27 genera were differentially represented in untreated core and peel tissues, which includes highly abundant genera such as *Aureobasidium, Penicillium, Alternaria* and *Sporobolomyces* (Figure 3B). In total, 15 of the 27 differentially represented genera were more abundant in core tissue than peel tissue, including *Alternaria* (10% vs. 4% of TR) and an unidentified genus in the family *Didymellaceae* (4% vs. 0.4% of TR). Conversely, a number of highly abundant genera were underrepresented in core tissue, including *Aureobasidium* (23% vs. 35% of TR)*, Penicillium* (6% vs. 35%), and *Sporobolomyces* (0.1% vs. 8% of TR).

Subsequently, the core and peel tissues were analyzed separately in order to examine the impact of the growing season on each tissue type independently. The growing season had a significant impact on the communities associated with untreated core tissues (R2 = 0.67, *p* < 0.001). Additionally, a higher Shannon diversity index (*p* < 0.05) was observed in untreated core tissue in 2019 than in the 2020 (Table 1). The 15 most abundant genera represented over 96% of the TR in core tissues in both 2019 and 2020 (Supplementary Figure S2A). However, significant shifts in many of these highly abundant genera were observed between growing seasons. Taxa such as *Aureobasidium* (37% vs. 8% of TR)*, Alternaria* (11% vs. 8% of TR) and an unidentified genus in the family *Didymellaceae* (7.5% vs. 0.3% of TR) had a significantly greater relative abundance in 2019, while genera such as *Cladosporium* (31% vs. 67% of TR) and *Penicillium* (3% vs. 12% of TR) were significantly overrepresented in 2020 relative to 2019. In all, 15 differentially represented genera by growing season were observed, with the majority of these genera being more abundant in 2020 (Figure 3C).

The growing season also accounted for significant variation in the fungal communities in untreated peel tissue (R2 = 0.74, *p* < 0.001), with a higher Shannon diversity index (*p* < 0.05) observed in these communities in 2019. The 15 most abundant genera accounted for over 97% of the total abundance in both the 2019 and 2020 growing seasons (Supplementary Figure S2B). However, in the peel tissue, fewer genera displaying differential representation by growing season were observed (Figure 3D); only five of these genera were observed, with *Penicillium* (8% vs. 61% of TR), *Cladosporium* (6% vs. 20% of TR), and *Colletotrichum* (0% vs. 1% of TR) all being less abundant in 2019, while *Sporobolomyces* (15% vs. 0.2% of TR) and an unidentified genus in the order *Saccharomycetales* (1% vs. 0% of TR) were more abundant in 2019 than in 2020.

### Effect of treatment on fungal communities in core tissue

Next, we investigated the impact of the treatment on the fungal communities present, and the potential influence of the growing season on the treatment effect. The growing season appears to have a greater influence on fungal community structure in core tissue (R2 = 0.6, *p* < 0.001) than the treatment effect does (R2 = 0.14, *p* < 0.001). A small but significant interaction effect was also observed between growing season and treatment (R2 = 0.09, *p* < 0.002). When all the treatments were considered together, the communities in core tissue formed distinct clusters based on the growing season according to the NMDS visualizations, with the communities sampled in 2019 associated with a significantly higher Shannon diversity index (*p* < 0.05) than those sampled in 2020 (Figure 1B; Table 1). The growing season also had a significant impact on community composition. A total of 23 differentially represented genera by growing season were identified, including highly abundant taxa such as *Cladosporium*, which was more abundant in 2019 (73% of TR in 2019 and 24% of TR in 2020, respectively) (Figure 3E). In contrast, *Aureobasidium* was less abundant in 2019 (6% of TR) compared to 2020 (39% of TR).

**Figure 4:**
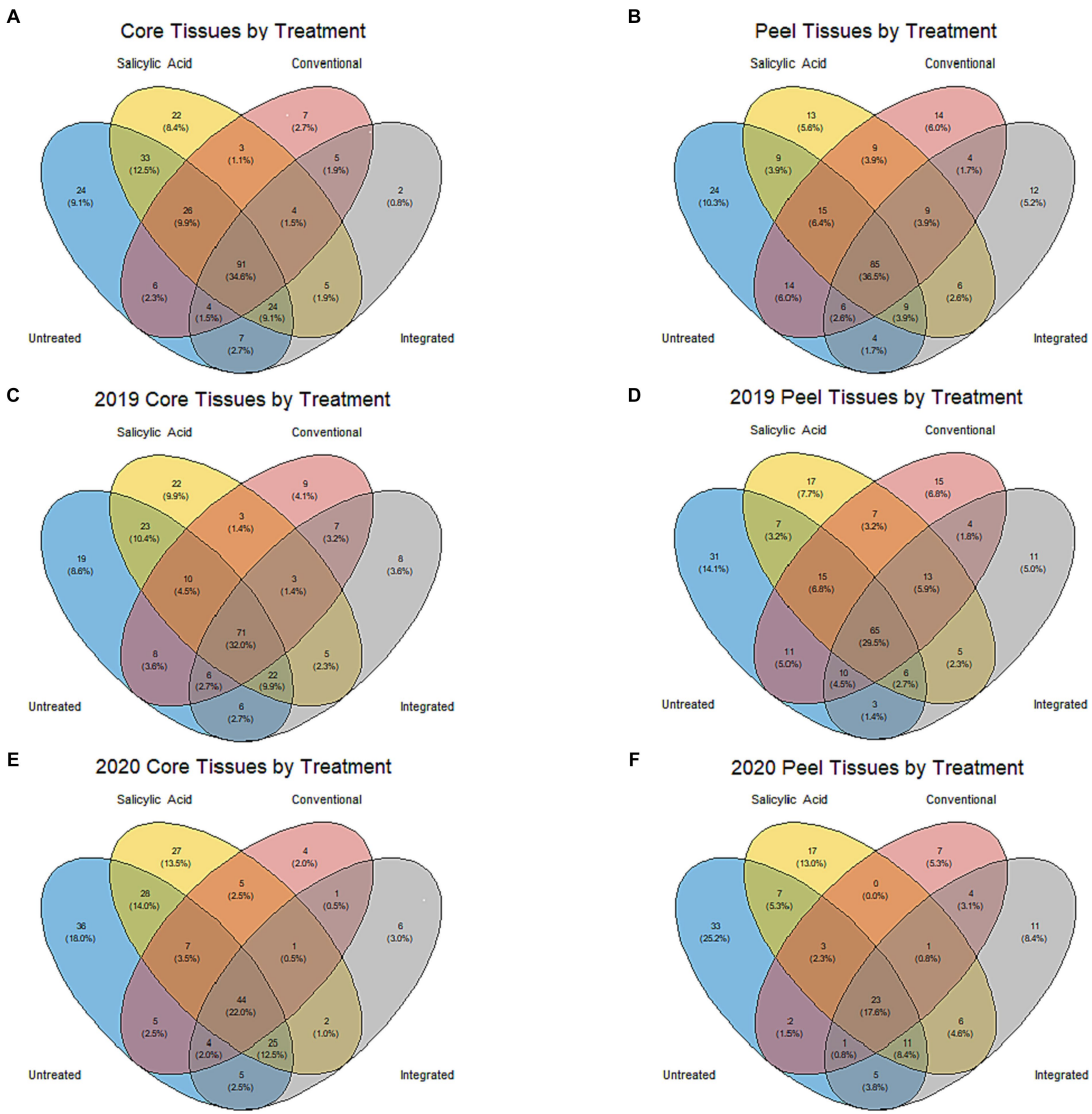
Venn diagrams showing individual fungal ASVs in different treatment groups. **(A)** Samples from core tissues (grouped based on treatment across both growing seasons). **(B)** Samples from peels grouped based on treatment across both growing seasons. **(C)** Samples from core tissues in the 2019 growing season. **(D)** Samples from peels in the 2019 growing season. **(E)** Samples from core tissues in the 2020 growing season. **(F)** Samples from peels in the 2020 growing season.

When the microbiomes from both growing seasons were combined in an NMDS visualization, the communities did not form distinct clusters by treatment, and no significant differences were observed in the Shannon diversity index values (*p* > 0.05) based on treatment (Figure 1B; Supplementary Table S2). A small number of genera dominated the fungal communities across all the treatments, with the top 15 genera accounting for over 95% of the total relative abundance in all treatment types (Figure 2C). A total of 12 genera with differential representation by treatment were identified (Figure 3F). The SA and control treatments were also associated with a greater number of ASVs, and 30% of ASVs were unique to these treatments, while the percentage of unique ASVs was much lower in the fungicide and integrated treatments (Figure 4A).

Given the influence of growing season on community composition, the data were separated and the impact of the treatments analyzed individually in each growing season to prevent the potential masking of the treatment effect. In the 2019 growing season, the treatment appeared to have a significant impact on variations in the community structure (R2 = 0.55, *p* < 0.001), although NMDS visualizations failed to generate clear clustering based on treatment (Supplementary Figure S3A). The Shannon diversity index (*p* > 0.05) for the communities in the 2019 growing season did not differ significantly by treatment (Supplementary Table S2). In total, 22 genera with differential representation by treatment were identified in 2019 (Figure 3G). Conventional fungicide use appeared to be a major factor influencing core community composition. For example, in 2019, *Cladosporium* was overrepresented in untreated tissue (31%) and tissue with the SA treatment (39%) compared to tissue with a conventional (13%) and integrated treatment (12%), while *Aureobasidium, Alternaria*, and *Penicillium* were overrepresented in the fungicide treatments when compared to SA-treated or untreated tissue. In the 2019 growing season, 29% of the total ASVs were unique to the SA-treated and/or untreated tissue, while 11% of ASVs were unique to the fungicide-treated tissues (Figure 4C).

In 2020, the treatment accounted for approximately 62% (*p* < 0.001) of community variation. According to an NMDS visualization, the microbiomes found in core tissue did not form distinct clusters by treatment (Supplementary Figure S3B). However, the communities in core tissue treated with conventional fungicide were characterized by significantly lower (*p* < 0.05) Shannon diversity index values than those in untreated or SA-treated tissue (Table 1). Only two differentially represented genera by treatment were identified in core tissue in 2020—*Taphrina* and *Genolevuria –* both of which account for less than 1% of the TR (Figure 3H). These two genera were apparently more abundant in fungicide-treated core tissue, with *Taphrina* being present only in very small proportions in tissue under the other treatment regimes (0.1% of the TR in untreated core tissue and 0.01% of the TR in SA-treated tissue). Only 6% of ASVs were unique to fungicide-treated core tissue in 2020, compared to 46% that were unique to untreated and SA-treated tissue (Figure 4E).

### Peel tissue

The growing season was the strongest factor affecting the fungal community structure of peel tissues (R2 = 0.41, *p* < 0.001), although the treatment also had a significant effect (R2 = 0.07, *p* < 0.013). An interaction effect was also observed between growing season and treatment (R2 = 0.12, *p* < 0.002). When visualized on an NMDS plot, the peel tissue fungal communities formed distinct clusters based on growing season and, in 2019, these communities were characterized by a significantly higher Shannon diversity index value (*p* < 0.05) than in 2020 (Figure 1C). Sixteen genera were differentially represented by growing season, including those with relatively high abundance such as *Penicillium*, which was more abundant in 2020 (68% of TR in 2020 vs. 22% of TR in 2019, respectively), and *Alternaria*, which was more abundant in 2019 (12% of TR in 2019 vs. 1% of TR in 2020, respectively) (Figure 3I).

Peel tissue fungal communities did not form distinct clusters by treatment when visualized by NMDS, and no significant differences in Shannon diversity index values were found based on treatment (Figure 1C; Supplementary Table S2). These communities were dominated by a small number of fungal genera, with the 15 most abundant ones accounting for over 98% of the total relative abundance across treatments (Figure 2D). Six differentially represented genera by treatment were identified, though none of these genera accounted for over 1% of the total relative abundance (Figure 3J). A higher percentage of unique ASVs was once again observed in untreated and SA treated tissue when compared to the fungicide treatments, and 20% of all ASVs were unique to untreated and SA treated peel tissue (Figure 4B).

Similarly to core tissue communities, the effect of the treatments on peel tissue communities was examined in each growing season independently to prevent masking of the treatment effect. The community composition of peel tissue was significantly impacted by the different treatment regimes (R2 = 0.33, *p* < 0.004) in the 2019 growing season. However, with NMDS visualization, these communities did not form apparent clusters by treatment and no significant differences were observed in their Shannon diversity index values by treatment (Supplementary Figure S3C; Supplementary Table S2). Five genera were differentially represented by treatment regime in this growing season (Figure 3K), but only one of these five genera accounted for over 1% of the total relative abundance (*Colletotrichum*), which was lower in untreated tissues (0.1% of TR) compared to tissues under the SA, conventional and integrated treatment regimes (2, 1.5 and 2% of TR respectively). The proportion of ASVs unique to fungicide-treated tissues was lower than the proportions unique to untreated or SA-treated tissues and, together, these unique ASVs accounted for approximately 13 and 25% of the total ASVs in the fungicide- and non-fungicide treated groups in 2019, respectively (Figure 4D).

Treatment had a significant impact on the community composition of peel tissue in the 2020 growing season (R2 = 0.35, *p* < 0.005), though these communities did not form distinct clusters based on an NMDS visualization (Supplementary Figure S3D). The communities subject to the conventional fungicide treatment had a lower Shannon diversity index value (*p* > 0.05) than those that had undergone the integrated treatment, but was similar to those of untreated or SA-treated samples (Table 1). Once again, five genera were differentially represented by treatment regime in this growing season (Figure 3L). Among these genera, both *Aureobasidium* (15% of TR in untreated tissues, 8% of TR in SA-treated tissues, 3% of TR in conventionally treated tissues, and 22% of TR in integrated treatments, respectively) and *Alternaria* (1% of TR in untreated tissues, 0.7% of TR in SA-treated tissues, 0.4% of TR in conventionally treated tissues and 2% of TR in integrated treatments, respectively) likely account for a total relative abundance of over 1%. The proportion of ASVs unique to the fungicide treatments was lower than those found in the SA and control treatments, and these ASVs likely account for approximately 17 and 44% of the total ASVs in the fungicide- and non-fungicide treatment groups in 2020, respectively (Figure 4F).

### Network analysis

**Figure 5:**
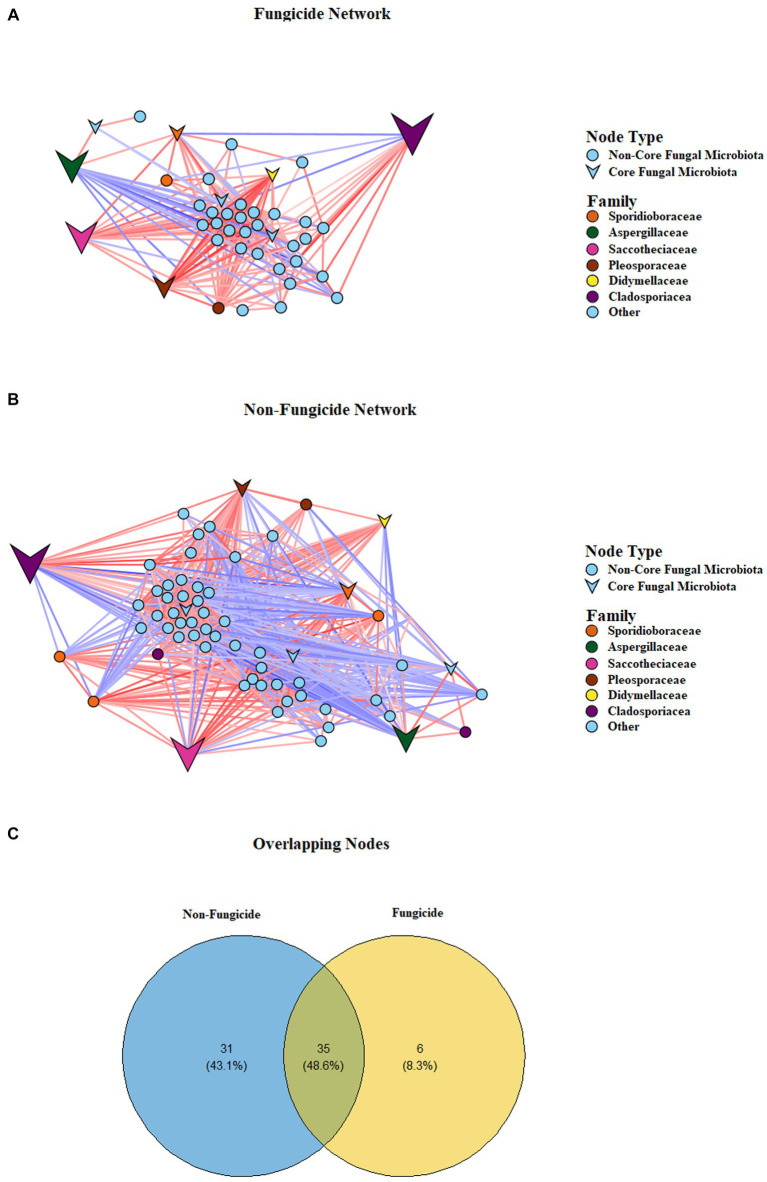
Correlation-based network showing potential interactions between fungal families. The lines (edges) connecting the nodes (fungal taxa) represent a positive (red) or negative (blue) co-occurrence relationship, and the intensity of the colour represents the strength of correlation. **(A)** Conventional and integrated treatments. **(B)** Untreated and SA treatments. **(C)** Venn diagram depicting node identity overlap and unique nodes in the two networks.

Given the differences in the composition of fungal communities and the presence of unique ASVs in the four treatment groups, we examined the impact of fungicides on the network characteristics of the apple fungal communities (Figures 5A,B). To assess network characteristics, samples were divided into two separate groups based on fungicide use: the non-fungicide network, which consisted of the untreated control and salicylic-acid-treated samples, and the fungicide network, which consisted of conventional fungicide and integrated treatments. The application of fungicides was associated with alterations in network complexity and connectivity. This was reflected in the network topological characteristics, as the number of nodes and interactions was lower in the fungicide-treated sample group than the SA-treated and control sample groups (Table 2). Furthermore, the non-fungicide network was associated with a larger network diameter and characteristic path length, as well as a higher average number of neighbours and overall network density. A total of 35 taxa were identified as nodes in both the non-fungicide and fungicide networks, while 31 and 6 taxa were unique to the non-fungicide and fungicide networks, respectively (Figure 5C). These nodes represented most of the highly abundant taxa in the dataset with a TR of over 98% in both networks.

**Table 2:** Global network characteristics of apple fungal communities.

Network (global) topological characteristics	Non-Fungicide	Fungicide
Number of nodes	66	41
Number of interactions	884	291
Average number of neighbours	26.79	14.20
Network diameter	5	3
Characteristic path length	1.61	1.53
Clustering coefficient	0.32	0.29
Network density	0.21	0.18

On the local (node) level, fungicide treatment was associated with lower values for the average number of neighbours (14.2 vs. 26.8, *p* < 0.01), neighbourhood connectivity (16.2 vs. 30, *p* < 0.001), and average shortest path length (1.2 vs. 1.5, *p* < 0.001), as well as a lower edge betweeness value (59.4 vs. 108.4, *p* < 0.001) (Table 3). In addition, the chemical fungicide treatments appeared to alter the balance of positive and negative interactions between community members. In the non-fungicide network, 44% of interactions were negative, compared to only 12% of all interactions in the fungicide network. Taken together, these results indicate a clear disruption of microbial cooperation in apple tissues treated with chemical fungicides (Figure 5; Table 3).

**Table 3:** Node (local) topological characteristics of apple fungal community networks.

Node (local) topological characteristics	Non-Fungicide	Fungicide
Degree***	13.40	7.10
Neighbourhood connectivity***	29.96	16.23
Average shortest path length**	1.48	1.23
Eccentricity*	2.56	2.05
Stress***	1895.41	335.37

The fungicide network was constructed using conventional and integrated treated samples, while the non-fungicide network was constructed with untreated and SA treated samples. *, **, and *** indicate *p* < 0.05, *p* < 0.01, and *p* < 0.001, respectively. Statistical significance was calculated via the Student *t*-test.

### Impact of treatments on disease incidence

Fruit was visually examined at harvest and after 4 months of cold storage in order to determine the impact of the different treatments on the incidence of fungal disease. In the 2019 samples, the incidence of apple scab was 22% in untreated control apple fruit, but was significantly reduced by SA treatment (10%, *p* < 0.05). Significantly reduced scab incidences of 4 and 5% were observed in the conventional and integrated treatments, respectively, (*p* < 0.05) (Supplementary Table S3). A similar pattern was observed with the incidence of black rot (5% untreated, 2% SA, 0.3% conventional and integrated, *p* < 0.05). In 2020, disease incidence was much lower at harvest compared to 2019. A significant difference was once again observed with apple scab, which had an incidence of 7% in untreated fruit, 4% in treated fruit, and approximately 2% in fruit with conventional and integrated treatments. The treatments were also associated with a significant decrease in post-harvest disease, with a substantial reduction in the incidence of black rot in 2019 (10% untreated, 3% SA, 1% conventional and 3% integrated, *p* < 0.05). A similar reduction in post-harvest disease incidence was not observed in 2020.

## Discussion

Chemical fungicides have been the primary means of fungal disease control for decades. However, increasing concerns over environmental, human and animal toxicity have led to greater restrictions on the use of these vital compounds (Ragsdale and Sisler, 1994; Steinhauer et al., 2018). Therefore, the development of new and environmentally friendly forms of disease control has been a focus of immense scientific interest. In particular, the maintenance of the microbiome has been cited as a potential eco-friendly means of disease control, particularly for the control of post-harvest fruit disease (Adam et al., 2016; Droby and Wisniewski, 2018). To implement microbiome maintenance for disease control a thorough understanding of how the available treatment options impact the fruit microbiome is needed. Although the literature has found evidence of variation in apple fungal communities between conventional and organic management practices, the impact of exogenous plant defence elicitors on these communities has yet to be explored (Abdelfattah et al., 2016; Bösch et al., 2021; Britt et al., 2021; McLaughlin et al., 2023). In addition to variation by treatment regime, spatial variation by tissue types and variations due to changes in environmental conditions between growing seasons have also been described (Abdelfattah et al., 2016; Bösch et al., 2021; Abdelfattah et al., 2021a).

### Variation between tissue types

This study provided evidence that the fungal communities associated with various types of apple fruit tissues respond differently to changes in environmental conditions between growing seasons. Individually, tissue type accounted for approximately 31% of the variation in the communities associated with untreated apples. Spatial variations in the microbiome by tissue type have been previously described, and previous research has suggested that the fungal community composition of these tissues could be partially determined by conditions within the tissue micro-environment (Abdelfattah et al., 2016, 2021a; McLaughlin et al., 2023). Indeed, 27 genera were differentially represented in untreated core and peel tissues in this study, including highly abundant genera such as *Aureobasidium, Penicillium, Alternaria* and *Sporobolomyces*, providing further evidence of the importance of the tissue micro-environment in determining the makeup of the fungal microbiome. Furthermore, given that the fungal communities of stem and calyx end of core tissues have commonly been associated with greater fungal diversities than those of peel tissues it seems apparent that these ecological niches may be more readily colonized, either due to morphological differences, increased accumulation of moisture, an easy entry point or increased nutrient availabilities within these tissues. Further investigation of the factors influencing the fungal communities of specific fruit tissues will be necessary to identify the most relevant factors. In our previous studies we have observed similar trends of high microbial diversity in the core tissues compared to peel tissues (Abdelfattah et al., 2021a; McLaughlin et al., 2023).

The 2020 growing season at our experimental orchard sites was characterized by significantly less precipitation than during 2019 (McLaughlin et al., 2023). The growing season had significant impacts on the apple microbiome, with the different environmental conditions in the 2 years, accounting for 42% of the variations in the fungal communities in untreated tissues. The presence of an interaction effect between tissue type and growing season (7%) suggests that tissue-specific micro-environments are affected differently by the macro-environmental conditions. When tissue-types are observed individually, growing season accounts for 67 and 74% of variations in the communities of core and peel tissues, respectively, providing further evidence that growing season impacts microbiome variation. On the whole, this corresponds to the observation in our previous study that the impact of the growing season was apparently greater on peel tissues than core tissues (McLaughlin et al., 2023). Questions including how altering the macro-environmental conditions can impact tissue-specific micro-environments, and how these specific micro-environmental changes in turn are reflected in the microbiome, are likely important considerations if microbiome maintenance is to be seriously considered in disease management applications.

### Variation between treatment regimes

The treatment regime accounted for approximately 14 and 7% of the variation in the fungal communities of core and peel tissues, respectively. Chemical fungicides do not specifically target only fungal pathogens, but also have a detrimental impact on resident fungal endophyte populations, leaving the host vulnerable to subsequent pathogen infection (Mohandoss and Suryanarayanan, 2009). Furthermore, in the absence of pathogen pressure, fungicide treatments such as seed coatings with systemic fungicides may actually be detrimental, and the elimination of beneficial endophytes and loss of microbials diversity may lead to associated losses in plant health and vigor (Ayesha et al., 2021). This is because beneficial endophytes contribute to plant growth and development by producing plant growth hormones, helping plants to absorb nutrients, water and protecting plants from both biotic and abiotic stresses. Notably, we observed similar characteristics in the microbiomes subjected to the chemical and integrated treatment regimes, despite the fact that the latter involved a large reduction in the use of chemical fungicides, suggesting that even a few applications of chemical fungicides can have immense consequences for the fruit microbiome. In core tissues especially, the conventional and integrated treatments were associated with a reduction in unique ASVs, as well as with a similar abundance of differentially represented genera. For example, *Cladosporium* was underrepresented in core tissues that underwent either conventional or integrated treatments, while *Aureobasidium, Alternaria, and Penicillium* were all overrepresented in these tissues in 2019 compared to in 2020. Conversely, the microbiomes of tissues treated with SA more closely resembled the microbiomes associated with untreated tissues, and these treatment regimes were associated with an increase in unique ASVs, with differentially represented genera generally having similar abundances in untreated and SA-treated tissue. This observation could be due to differences in the treatment regimes’ modes of action. Whereas chemical fungicide applications in both the conventional and integrated treatment regimes may eradicate both pathogens and resident endophytes in apple fruit, SA applications would instead induce systemic acquired resistance and prime plant defences against future colonization (Ward et al., 1991). However, in our current study, the incorporation of SA in a conventional spray program did not appear to greatly alter the impact of chemical fungicides on the fungal microbiota of apple fruit at harvest. Nevertheless, it is important to emphasize that a very limited amount of information about the effect of incorporation of SA application in conventional apple production is currently available and more comprehensive analysis of the use of SA in combination with chemical fungicides is necessary. Given that the SA treatments occurred earlier in the summer, and the final treatment of both regimes was with a chemical fungicides. The lack of major differences in the fungal microbiota observed in apple from the integrated and conventional spray programs at harvest could potentially be explained by the timing of fungicide applications. Thus, a future study on the examination of the fungal microbiota earlier in the growing season and directly following SA or Fungicide application may reveal greater differences between the two regimes.

As was observed in the various tissue types, the fungal microbiomes appeared to respond differently under different treatment regimes to changes in the macro-environment between growing seasons, and the interaction between treatment regime and growing season may account for approximately 9% of the variations in fungal communities in cores and 12% in peels, respectively. In addition, the effect of the various treatment regimes on fungal communities was apparently greater in 2019 in both core and peel tissues, suggesting that the treatment effect has a greater impact under conditions with greater microbial diversity — in this case, the “wet” summer of 2019. Nevertheless, the impact of treatment regimes on the fungal microbiome is not static and is influenced by the environmental conditions under which these treatments are applied. Thus, in order to actively manipulate the apple fruit microbiome for disease control purposes, a thorough understanding of the impacts of treatments on the microbiome of apple fruit under different environmental conditions will be necessary.

### Network characteristics

The development of dysbiosis – or an imbalance in the composition of the microbiome – has been associated with increased vulnerability to disease. For example, conditions which resulted in a loss of microbial diversity in mango fruit were correlated with the increased development of stem-end rot (Diskin et al., 2017). Therefore, it has been proposed that the comparison of the diversity and structure of microbial communities could be beneficial in predicting their susceptibility to disease. However, analyses of microbial diversity and structure tend to be simplistic and cannot adequately describe how stressors impact the microbial community (Shade, 2017). Evidence suggests that the stability, population dynamics and function of microbial communities are heavily influenced by a network of microbe-microbe interactions, and therefore shifts in the relative abundance of an individual microbe can also have profound impacts on other members of the network (Czárán et al., 2002; Kinkel et al., 2011; Friedman et al., 2017; Banerjee et al., 2018). In addition, the ratio between competitive and cooperative interactions in a network can also be a useful predictor of microbial community stability, and increased cooperation in a network may lead to the destabilization of the microbial community due to the establishment of positive feedback loops and co-dependency (Coyte et al., 2015). Therefore, the investigation of microbial cooperation has emerged as a higher resolution method of evaluating the impact of various stressors on microbial communities (Karimi et al., 2017; Schlatter et al., 2018; Esan et al., 2019).

In this study, we demonstrated that the use of chemical fungicides is associated with a reduction in network complexity and the disruption of microbial cooperation. While the use of chemical fungicides was not always associated with a reduction in the Shannon diversity index in this study, it was associated with a loss in microbiome stability. This corresponds well with previous observations that tissues treated with chemical fungicides may be more vulnerable to subsequent pathogen infections due to the eradication of endophytic communities (Mohandoss and Suryanarayanan, 2009). Intriguingly, the most impactful nodes, defined as the nodes that had the highest number of interactions (top 10%) in the network, differed in each network, and the majority of these impactful nodes were low in abundance (< 1% of the total reads). In the fungicide-treated network, these nodes corresponded to the genus *Alternaria* (25 unique interactions), as well as the genera *Muriphaeosphaeria, Acremonium*, an unidentified genus in the family *Phaeosphaeriaceae*, and *Setomelanomma* (all with 22 interactions). Among these nodes, only *Alternaria* was highly abundant in this network. In contrast, in the non-fungicide network, the most impactful nodes corresponded to the genera *Alternaria* (42 interactions), *Gelidatrema* (41 interactions), as well as *Penicillium, Neocucurbitaria, Muriphaeosphaeria* and an unidentified taxon in the family *Phaeosphaeriaceae* (all with 40 interactions). Two of these nodes were also very abundant, with unidentified species in the *Alternaria* and *Penicillium* genera accounting for around 6 and 19% of the total reads, respectively. As such, nodes did not have to be highly abundant to be impactful, since nodes that were low in abundance accounted for a majority of the most connected and impactful nodes in each network. Consequently, the loss of species that were originally low in abundance through chemical fungicide treatments can clearly have profound implications for overall microbial cooperation in fruit tissue communities, although this loss may not be obviously reflected in their Shannon diversity indices.

How the most abundant families (> 1% of TR) within the networks interacted with one another was particularly interesting, and these dynamics were relatively stable between the networks. For example, in the non-fungicide network, *Cladosporiaceae* was the most abundant family, and the node belonging to this family had negative interactions with all members of the *Sporoidiobolaceae* family and positive interactions with the *Pleosporaceae* family but had no direct influence on the abundance of *Saccotheciaceae* or *Aspergillaceae*. These same dynamics were also observed in the fungicide network. As changing relationship dynamics between the networks cannot explain shifts in the relative abundance of these highly abundant families, it is clear that they instead may occur partially as a result of the extinction of highly connected nodes and a reduction in microbial interactions in the fungicide network.

### Disease incidence

In this study, we provide evidence that lower microbial diversity is not necessarily a predictor of disease development. Disease incidence was greater in the growing season of 2019, when apple fungal communities were characterized by significantly higher Shannon diversity index values, than in 2020. This could be attributed to the significantly greater precipitation in 2019 compared to 2020. Since many fungal pathogens are dependent on splashing rain and lengthy periods of high humidity for dispersal and infection, the dry growing season in 2020 may not have been conducive to the success of these pathogens (Madden, 1997; Chiu et al., 2022). Thus, the reduction in microbial diversity, possibly due to unfavorable environmental conditions for apple-associated microbiota, was not a predictor of increased disease susceptibility, as the same environmental pressures were applied to the fungal pathogens. Therefore, it is apparent that the Shannon diversity index may not be a reliable predictor of disease susceptibility, since other factors, like environmental conditions, may be more significant determinants of successful infection.

Similarly, here we presented evidence that the disruption of microbial cooperation by fungicide treatments is not associated with increased disease incidence in apple fruit, either at harvest or post storage. Despite a steep decrease in unique ASVs and a loss in network complexity under the fungicidal treatment regimes relative to the non-fungicide treatment regimes, the lowest incidence of apple scab, black rot, and bitter rot was observed in the fungicide treatment regimes. The efficacy of the fungicide treatment regimes, despite their associated disruption of the fruit microbiome, could be explained by these pathogens’ infection periods, which tend to occur earlier in the growing season when these treatments are still being actively applied. For example, apple fruit is most susceptible to *Venturia inaequalis*, the pathogen responsible for apple scab, early in its development and becomes less susceptible as it matures (Schwabe et al., 1984). In addition, early fungicide applications, particularly during bud burst, have been proven to be essential in controlling the levels of the primary inoculum of *Colletotrichum acutatum*, one of the species responsible for bitter rot, thus preventing secondary infections throughout the growing season, while later fungicide applications have been shown to be less effective in disease protection (Everett et al., 2018). Consequently, although the disruption of fungal communities by chemical fungicides may make communities more vulnerable to subsequent infections, these infections do not materialize by the time fungicide application has ceased and the apples are being harvested, since the infection window has passed. Furthermore, the integrated treatment performed similarly to the full conventional treatment both at harvest and post storage. This is a first report showing that the integrated treatment of SA and chemical fungicides could provide effective disease control in apple, with a significant reduction in the use of conventional fungicides, and suggesting that the integration of SA or other plant defence elicitors may be a promising method of reducing conventional fungicide use. Conversely, the co-application of plant defence elicitors mixed with chemical fungicides can also improve disease control, particularly in cases where the pathogen is prone to resistance. For instance, the inclusion of Actigard^®^ (an SA analogue) into conventional spray programs increased the efficacy of several chemical fungicides against tobacco blue mould (LaMondia, 2008), while combining strobilurin with acibenzolar-S-methyl allows for the effective control of white rust in spinach without the induction of leaf chlorosis (Leskovar and Kolenda, 2002). As such the integration of plant defence elicitors into conventional programs could significantly contribute to a reduction in fungicide, or instead be used to improved fungicide efficacy. An in-depth evaluation of the effects of the incorporation of SA or other plant defence elicitors into conventional fungicide spray programs for pre and post storage disease control is necessary in order to determine the broader feasibility of these strategies.

## Conclusion

Maintaining a healthy or balanced fruit microbiome may have significant potential in terms of biocontrol of fruit pathogens, especially post-harvest. Numerous factors, including tissue type, environmental conditions, treatment regimes, genetic makeup, and geographical location have all been demonstrated to significantly influence the makeup of the fungal microbiome. This study observed significant differences in the fungal communities of Honeycrisp apples in Nova Scotia depending on the tissue type and treatment regime (non-fungicidal or fungicidal), also demonstrating interactions between these variables and changes in environmental conditions between growing seasons. Furthermore, this research underscores the significant influence of chemical fungicide treatments on fungal communities and community networks, and provides evidence that non-eradicative treatments, such as the plant defence elicitor, SA, are less disruptive to the apple fruit microbiome. Finally, the evidence presented in this manuscript suggests that incorporating SA in conventional spray programs might be a promising means of reducing conventional fungicide usage while maintaining effective disease control in apple.

## Data availability statement

The datasets generated in the current study are available in the [SRA NCBI] repository, and can be accessed at the following link: https://www.ncbi.nlm.nih.gov/sra/PRJNA1021219.

## Author contributions

MM: Data curation, Formal analysis, Investigation, Methodology, Writing – original draft, Writing – review & editing. SY: Data curation, Formal analysis, Writing – review & editing. PA: Funding acquisition, Project administration, Resources, Writing – review & editing. SA: Conceptualization, Data curation, Formal analysis, Investigation, Methodology, Funding acquisition, Project administration, Resources, Supervision, Validation, Writing – review & editing.

## Footnotes

^1^ https://imr.bio
