# Supplementary material for: The effects of chemical fungicides and salicylic acid on the apple microbiome and fungal disease incidence under changing environmental conditions
Source: Front Microbiol. 2024 Feb 5;15:1342407. doi: 10.3389/fmicb.2024.1342407 (PMC10875086; doi:10.3389/fmicb.2024.1342407)
Supplement: Supplementary file 1 [file Data_Sheet_1.PDF]

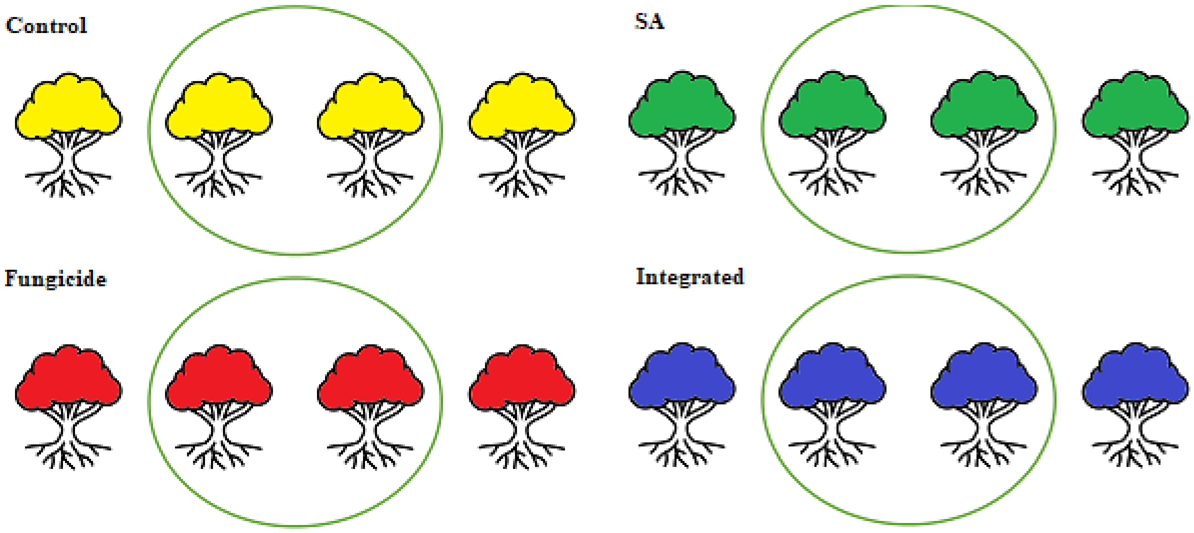

SUPPLEMENTARY FIGURE 1

Experimental Design of the treatments. 64 apple trees were divided into four blocks of 16 trees, each undergoing one of four treatments in a randomized complete block design with four replicates. The four treatments were as follows; (i) conventional (chemical fungicides), (ii) integrated (chemical fungicides and SA), (iii) SA alone, and (iv) control (untreated). The SA treatment consisted of 12 (2019) and six (2020) foliar sprays of 1-mM SA during the growing season, while the conventional treatment consisted of 12 foliar sprays of chemical fungicides during both growing seasons. The integrated treatment consisted of a combination of six applications of a 1-mM SA spray and six separate applications of chemical fungicides each year. To prevent the effect of drift between treatment groups, apple fruits for microbiome analysis were sampled only from the centre 2 trees of each treatment block.

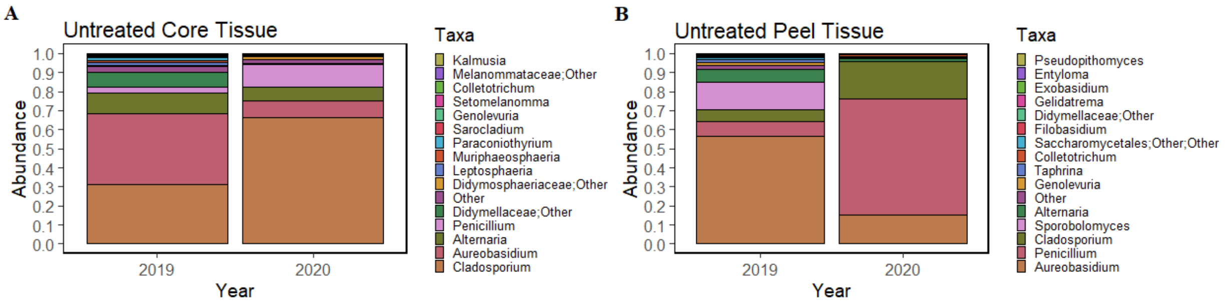

SUPPLEMENTARY FIGURE 2 Relative abundance of the top 15 most abundant genera in Honeycrisp apples. **A,** Communities in samples of untreated core tissues grouped by growing season. **B,** Communities in samples of untreated peel tissues grouped by growing season.

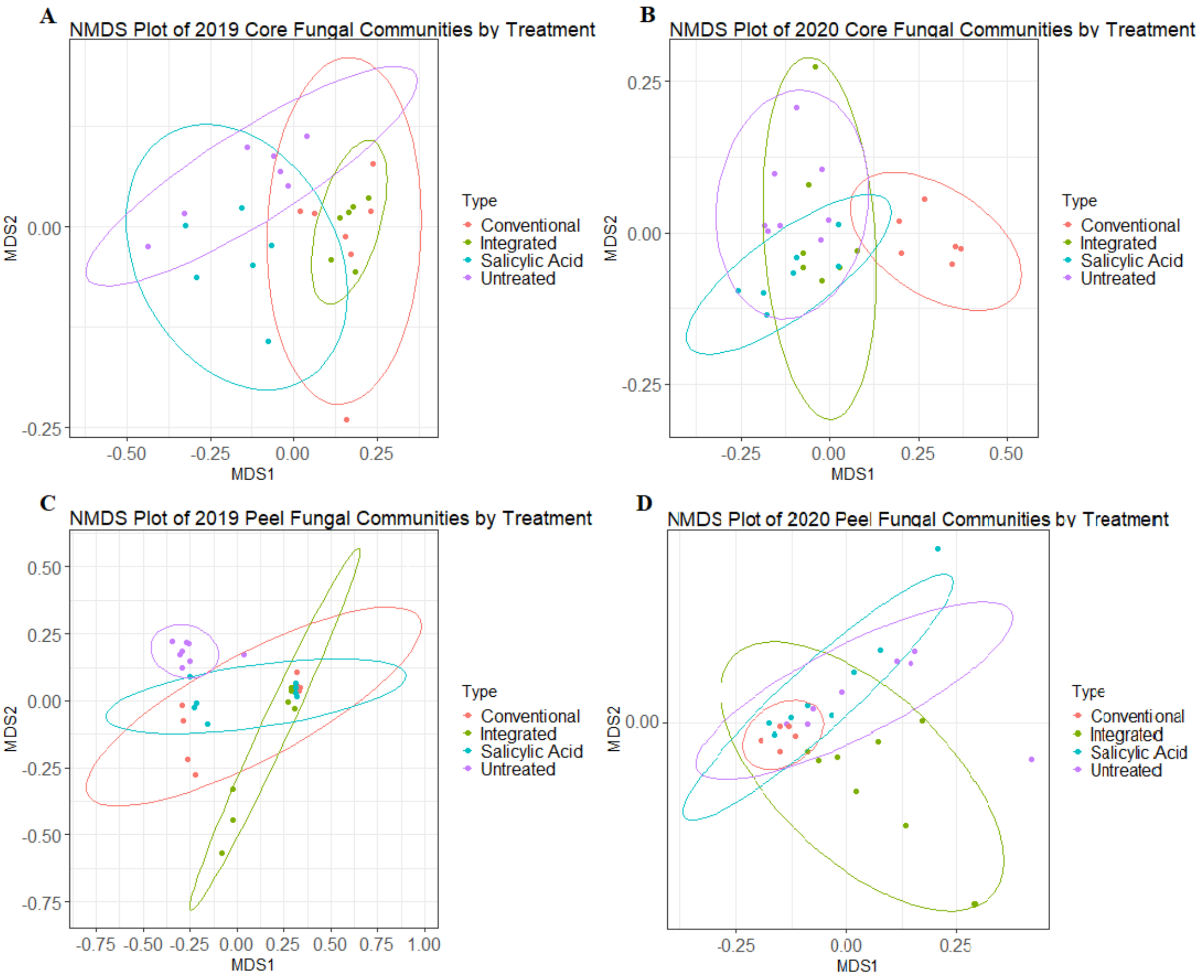

SUPPLEMENTARY FIGURE 3 Non-metric multidimensional scaling (NMDS) of the fungal communities of Honeycrisp apples at the ASV level. **A,** The communities in 2019 core tissues clustered by treatment regime. **B,** The communities in 2020 core tissues clustered by treatment regime. **C,** The communities in 2019 peel tissues clustered by treatment regime. **D,** The communities in 2020 peel tissues clustered by treatment regime.
